# Convergent and divergent gray matter volume abnormalities in unaffected first-degree relatives and ultra-high risk individuals of schizophrenia

**DOI:** 10.1038/s41537-022-00261-9

**Published:** 2022-06-04

**Authors:** Bei Lin, Xian-Bin Li, Sen Ruan, Yu-Xin Wu, Chao-Yue Zhang, Chuan-Yue Wang, Lu-Bin Wang

**Affiliations:** 1 The Brain Science Center, Beijing Institute of Basic Medical Sciences, Beijing, 100850 China; 2 The National Clinical Research Center for Mental Disorders & Beijing Key Laboratory of Mental Disorders, Beijing Anding Hospital, Capital Medical University, Beijing, 100088 China

**Keywords:** Biomarkers, Schizophrenia

## Abstract

High-risk populations of schizophrenia can be mainly identified as genetic high-risk based on putative endophenotypes or ultra-high-risk (UHR) based on clinically manifested symptoms. Previous studies have consistently shown brain structural abnormalities in both genetic high-risk and UHR individuals. In this study, we aimed to disentangle the convergent and divergent pattern of gray matter alterations between UHR and unaffected first-degree relatives from genetic high-risk individuals. We used structural MRI scans and voxel-based morphometry method to examine gray matter volume (GMV) differences among 23 UHR subjects meeting the Structured Interview for Prodromal Syndromes (SIPS) criteria, 18 unaffected first-degree relatives (UFDR), 26 first-episode schizophrenia patients (FES) and 54 healthy controls (CN). We found that a number of brain regions exhibited a monotonically decreasing trend of GMV from CN to UFDR to UHR to FES. Compared with CN, the UHR subjects showed significant decreases of GMV similar to the patients in the inferior temporal gyrus, fusiform gyrus, middle occipital gyrus, insula, and limbic regions. Moreover, the UHR transformed subgroup had significantly lower GMV than UHR non-transformed subgroup in the right inferior temporal/fusiform gyrus. On the other hand, the UFDR subjects only showed significant GMV decreases in the inferior temporal gyrus and fusiform. Moreover, we found GMV in the occipital lobe was negatively correlated with the UHR subjects’ composite positive symptom of SIPS, and GMV in the cerebellum was positively correlated with FES subjects’ symptom severity. Our results suggest that GMV deficits and regional dysfunction are evident prior to the onset of psychosis and are more prominent in the UHR than the UFDR individuals.

## Introduction

Schizophrenia is a severe chronic psychiatric disease characterized by diverse psychopathology features, which include positive symptoms (delusions and hallucinations), negative symptoms (impaired motivation, reduction in spontaneous speech and social withdrawal), and cognitive impairments^1^. Early identification and intervention during high-risk period are considered as effective precautionary measures in the forefront of schizophrenia research^2,3^. High-risk populations of schizophrenia can be mainly identified as genetic high-risk based on putative endophenotypes^4–6^ or ultra-high risk (UHR) based on clinically manifested symptoms^7^. The determination of structural biomarkers predicting susceptibility to psychosis has the potential in filling the gap between basic and clinical neuroscience for the diagnosis and treatment of schizophrenia^8^.

The commonly prodromal period preceding the onset of schizophrenia is considered as clinical high risk (CHR) or at-risk mental state (ARMS) or UHR for psychosis, which focuses on individuals presenting with subthresholded psychotic features^9^. Follow-up studies have shown that about 20–35% of the UHR individuals convert to frank psychotic disorder within the first 2 years^10^. Previous structural MRI studies have found that the gray matter volume (GMV) decline in the UHR individuals appear to be qualitatively similar but less severe to the abnormalities in schizophrenia patients, particularly in the insula, hippocampus, and wide frontal and temporal regions^11–13^. Moreover, reduced GMV in the frontal and temporal regions was found to be associated with the severity of psychotic symptoms and global functioning in the UHR individuals^14^. Several follow-up studies have further indicated that the UHR individuals who exhibit more severe structural deficits at baseline tend to have a higher risk of conversion subsequently^15–17^.

On the other hand, schizophrenia has a strong genetic basis. The concordance rate of monozygotic twins is 40–50% in their lifetime^18^, and the risk of other first-degree relatives of schizophrenia patients is about 10 times higher than that of general population^19^. A study of twins concordant and discordant for schizophrenia indicated that GMV reductions in frontal and temporal lobe, sensory-motor cortices, insula, hippocampus, and cerebellum are related to the genetic risk of schizophrenia^4,20^. Researches of non-twin biological first-degree relatives also provide evidence for genetic-liability effects on the disease-related gray matter abnormalities in schizophrenia^21^. As mentioned in the previous literatures, unaffected first-degree relatives (UFDR) of schizophrenia are shown to share some structural brain abnormalities with patient probands in several frontal and temporal regions, with a significant regional volume reduction intermediate to those observed in patients and controls^21,22^. These studies suggest that the morphological alterations in patients with schizophrenia are at least partly attributable to genetic factors related to the illness^20^.

Despite the accumulating evidence, findings remain unclear in terms of which and to what degree brain abnormalities in schizophrenia are attributable to genetic vulnerability factors or clinically manifested symptoms. To date, only a few studies evaluate brain abnormalities in both genetic high-risk and UHR subjects in comparison with patients with schizophrenia and normal controls. An event-related functional MRI study has proven that functional deficits associated with spatial working memory processing emerge in the UHR subjects before the onset of schizophrenia and compensatory neural processes exist in the subjects with genetic liability to schizophrenia^23^. Another study compared GMV and resting-state local brain activity differences among five subjects including the normal control, the genetic high-risk, the UHR, the first-episode, and chronic schizophrenia^24^. However, this study only observed significantly reduced GMV in the bilateral occipital lobe in the genetic high-risk group, but failed to find the abnormality of GMV in the UHR subjects, nor the change of resting brain activity in the UHR and genetic high-risk subjects.

In this study, we applied voxel-based morphometry (VBM)^25,26^ to examine subtle and extensive changes in characteristics of brain morphology between schizophrenia high-risk subjects defined by clinically manifested symptoms and genetic risk. Specifically, by examining the GMV of the UHR subjects, the UFDR, the first-episode schizophrenia (FES), and the control subjects, we aimed to disentangle the convergent and divergent pattern of gray matter alterations between the UFDR and the UHR subjects. Because the UHR subjects has already presented some subthresholded psychotic symptoms, we speculate that the structural abnormalities of the UHR subjects would be more severe than that of the UFDR subjects. We also hypothesized that there would be a correlation between the GMV of specific brain regions and symptom severity of the UHR and FES individuals.

## Materials and methods

### Subjects

We recruited 121 subjects from Beijing Anding Hospital and the local community (Table 1). The FES group consisted of 26 clinically stable patients, who were diagnosed based on assessment with the Structured Clinical Interview for the Diagnostic (SCID) and Statistical Manual of Mental Disorders-IV (DSM-IV) and had symptoms for less than 12 months. The Positive and Negative Symptom Scale (PANSS)^27^ was used to assess FES patients’ symptoms. The onset age of the FES patients was 21.23 ± 5.88 years, and the average illness duration from onset to pre-scan was 2.07 ± 1.79 years. The UHR group consisted of 23 subjects, who were assessed with the Structured Interview for Prodromal Syndromes (SIPS)^28^ and had composite positive symptom scores of no less than 3. The UFDR group was composed of 18 subjects who were clinically asymptomatic based on SIPS assessment, and had at least one first-degree relative with schizophrenia. The healthy control (CN) group consisted of 54 health volunteers, who had no lifetime or family history of any psychiatric disorder. Other criteria for selecting the subjects were as follows: right-handed, native Chinese speakers, no MRI contraindications, no major head trauma, no history of substance dependence, and no history of neurological disorder or other psychiatric illness. Written informed consent was obtained from all the subjects prior to data collecting and brain scanning. The experimental procedure was approved by the research ethics committee of Beijing Anding Hospital and conducted in accordance with the Declaration of Helsinki.

**Table 1:** Demographic and clinical characteristics of the subjects.

Characteristic	CN	UFDR	UHR	FES	*P* (T/F; Df)
Sample size	54	18	23	26	-
Gender (M/F)	34/20	5/13	18/5	17/9	<0.05 (11.55; 3)
Age (years)	25.9 ± 4.6	23.9 ± 4.8	23.0 ± 5.8	24.0 ± 6.4	0.105 (2.09; 3,117)
Education (years)	12.6 ± 3.2	14.1 ± 2.7	13.1 ± 3.1	12.6 ± 3.2	0.266 (1.34; 3,117)
SIPS					
Positive	-	1.1 ± 1.6	9.1 ± 2.7	-	<0.001 (10.11; 32)
Negative	-	2.5 ± 4.3	10.3 ± 4.5	-	<0.001 (5.19; 32)
Disorganization	-	0.9 ± 1.8	5.1 ± 2.6	-	<0.001 (5.44; 32)
General	-	1.4 ± 2.2	5.2 ± 3.4	-	0.001 (3.91; 32)
PANSS total	-	-	-	90.9 ± 14.4	-
Positive	-	-	-	24.9 ± 5.6	-
Negative	-	-	-	23.8 ± 7.8	-
General	-	-	-	42.3 ± 6.9	-

SIPS information was not available for 2 and 5 UFDR and UHR subjects, respectively. PANSS information was not available for 5 FES subjects.

At the time of enrollment, 11 FES patients were receiving antipsychotics, including risperidone (*n* = 5), paliperidone (*n* = 2), olanzapine (*n* = 1), haloperidol (*n* = 1), aripiprazole (*n* = 1) and quetiapine (*n* = 1). Besides, 3 UHR subjects in this study were medicated with antipsychotics, including risperidone (*n* = 1), olanzapine (*n* = 1) and amisulpride (*n* = 1). And 1 UHR subject was receiving anti-depressant (fluvoxamine). The average daily dose of antipsychotics, factoring in olanzapine equivalent based on ref. ^29^, was 10.59 ± 5.34 and 9.67 ± 8.96 mg in the medicated FES patients and UHR subjects, respectively.

### Data acquisition

The MRI scans were performed at the Imaging Center for Brain Research, Beijing Normal University using a Siemens Trio 3-T scanner with a standard 12-channel head coil (Siemens, Erlangen, Germany). A standard radiofrequency head coil was used with foam padding to restrict head motion. A 3D T1-weighted Magnetization Prepared Rapid Gradient Echo Imaging (MPRAGE) sequence was used to acquire high-resolution anatomical scans with the following parameters: 176 sagittal slices, repetition time (TR) = 2530 ms, echo time (TE) = 3.45 ms, flip angle (FA) = 7°, slice thickness = 1 mm, acquisition matrix = 256 × 256, in-plane resolution = 1 × 1 mm^2^. Other MRI modalities were not used in the current study and therefore were not described here.

### Image processing

Image processing was performed by VBM8 toolbox (http://www.dbm.neuro.uni--jena.de/vbm8/) of the SPM12 software (Wellcome Department of Imaging Neuroscience, University College London, UK; http://www.fil.ion.ucl.ac.uk/spm) running on MATLAB 2013b. First, the raw MRI data were checked by visual inspection to ensure no obvious artifacts. Then, the structural MRI images of all the subjects were segmented into gray matter, white matter, and cerebrospinal fluid in native space. The resultant gray matter segments were spatially aligned to a high-dimensional Diffeomorphic Anatomical Registration Through Exponentiated Lie Algebra (DARTEL) template and normalized to the MNI space. Finally, the modulated normalized gray matter images were smoothed with a 6 mm FWHM Gaussian kernel.

### Statistical analysis

One-way voxel-wise ANOVA was performed to compare GMV differences between the CN, UFDR, UHR, and FES groups. Given the difference in males/females for the UFDR group, sex was used as a covariate in the ANOVA model. Subsequently, post hoc Bonferroni multiple comparisons were conducted for the significant clusters by using two-sample t-test, to reveal the abnormal pattern of GMV in different clinical groups. Next, to provisionally evaluate the clinical significance of GMV abnormality, we correlated it with the PANSS scores (total, positive and negative) for the FES subjects and the SIPS scores (positive and negative) for the UHR subjects, respectively. Voxels with GMV below 0.2 were excluded from the statistical analysis, to reduce the potential for misclassification of voxels, especially with regard to gray matter boundaries. The significance level was set at *P* < 0.005 at the voxel level. A family-wise error (FWE) method was employed to correct for false positives due to multiple comparisons (*P*_FWE_ < 0.05) at the cluster level.

## Results

### GMV differences across groups

**Fig. 1:**
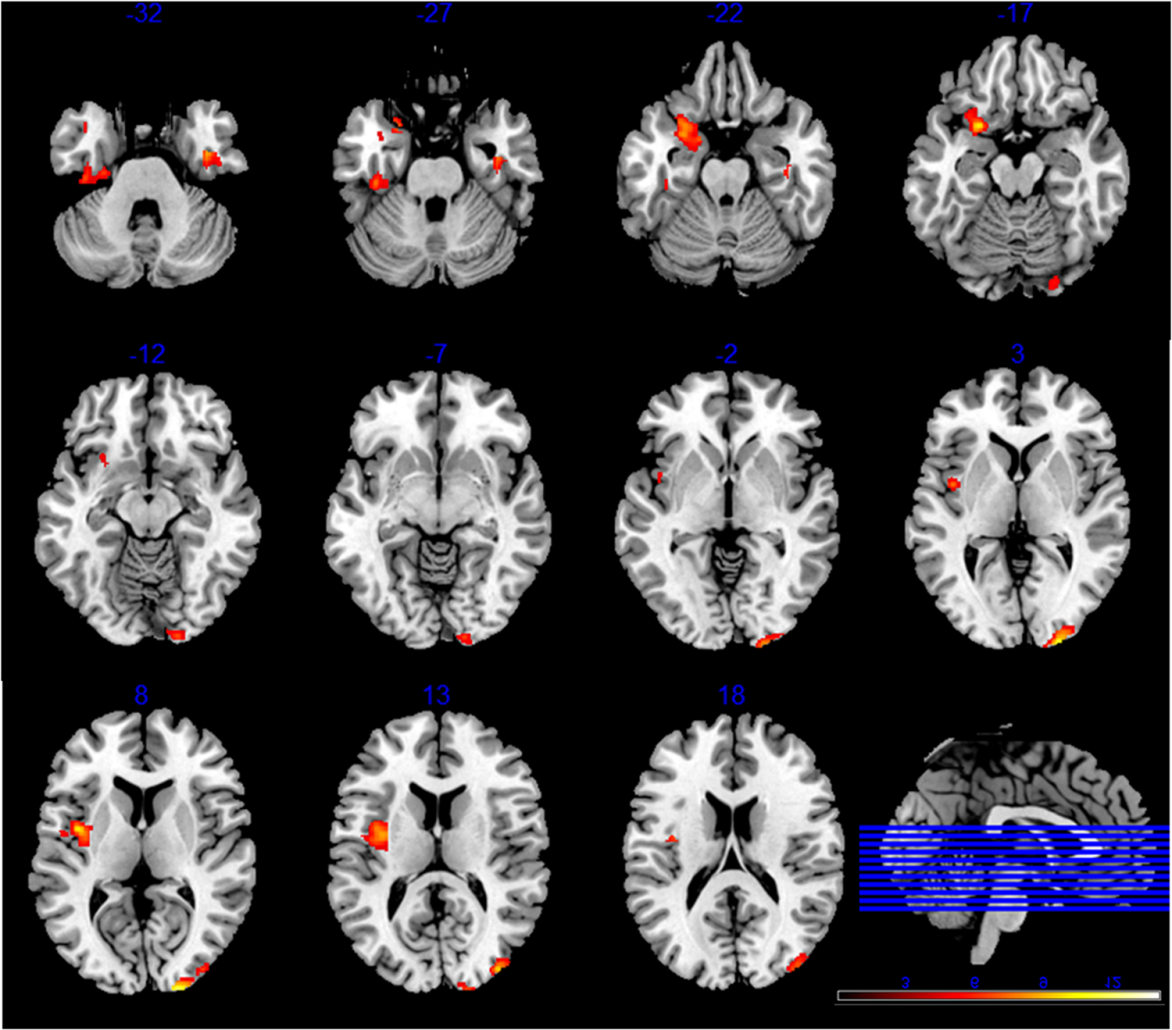
One-way ANOVA analysis shows differences in GMV across the four groups (CN, UFDR, UHR, and FES). The statistical map was thresholder at *P*_voxel-wise_ < 0.005 (uncorrected), then corrected for multiple comparisons to *P*_FWE_ < 0.05 by using a cluster threshold (*k* > 691 voxels or 2332.125 mm^3^). Color bars represent the *F*-value.

**Fig. 2:**
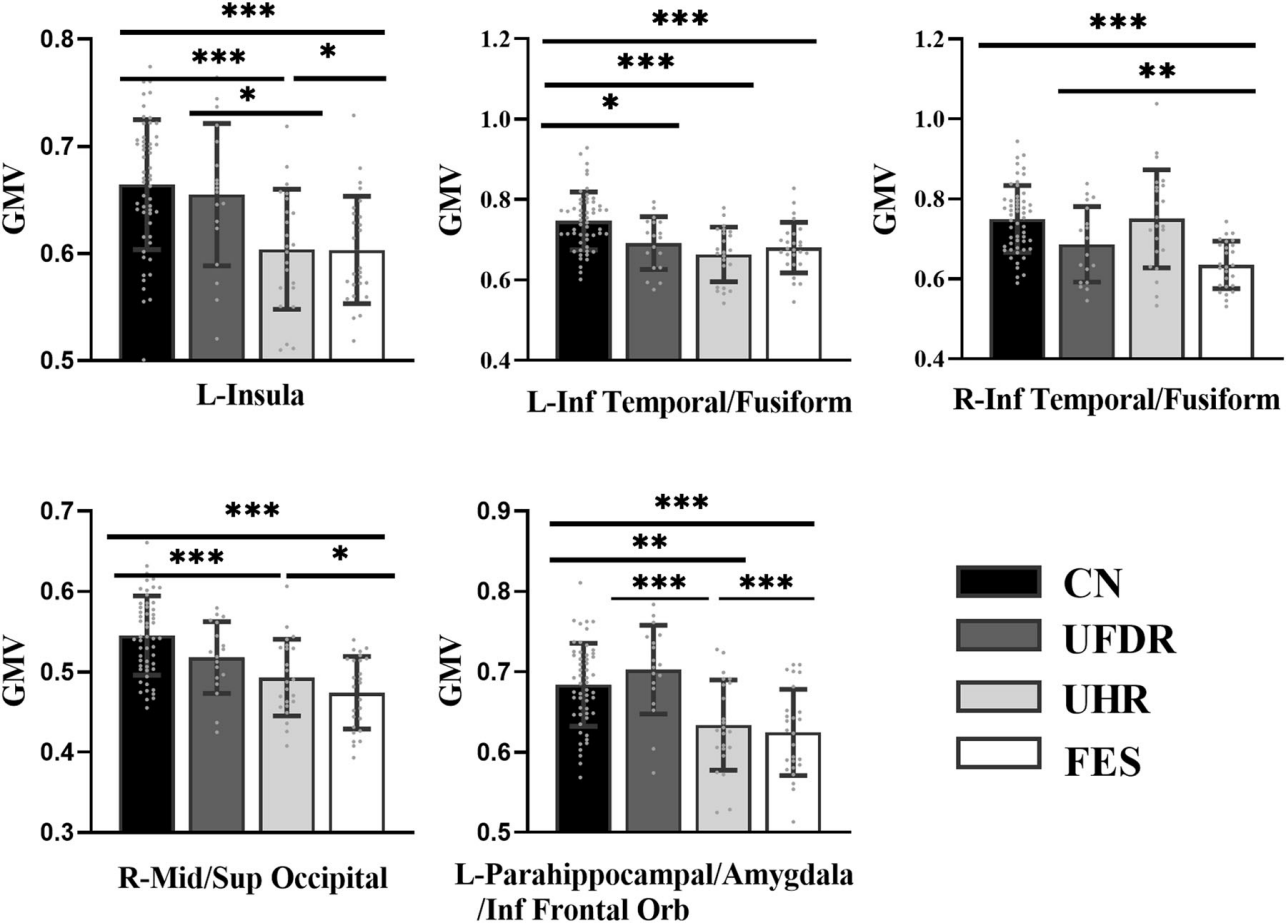
Brain regions show differential GMV across the four study groups. To show pairwise between-group GMV differences, significant clusters resulting from one-way ANOVA across the four groups were analyzed by post hoc Bonferroni multiple comparisons. Asterisk, ***P ≤ 0.001, **P ≤ 0.01, *P ≤ 0.05. Gray Scatter plot indicates the GMV of subjects. Error bars indicate the standard error of the mean. Inf Temporal, inferior temporal gyrus. Mid/Inf Occipital, Middle/Inferior occipital gyrus. Inf Frontal Orb, orbital frontal cortex.

One-way ANOVA analysis revealed widely distributed brain regions that showed significant GMV differences among the four groups (*P*_FWE_ < 0.05, see Fig. 1). For all these regions, the GMV was markedly reduced in the FES patients than in the CN subjects (Cohen’s d: 0.98–1.58). Compared with the CN subjects, the FES patients exhibited GMV decline in the left insula (CN: 0.66 ± 0.06, FES: 0.60 ± 0.05, *T*_78_ = 4.37, *P* < 0.001), bilateral inferior temporal/fusiform gyrus (left, CN: 0.75 ± 0.07, FES: 0.68 ± 0.06, *T*_78_ = 4.06, *P* < 0.001; right, CN: 0.75 ± 0.08, FES: 0.64 ± 0.06, *T*_78_ = 5.37, *P* < 0.001), right middle/superior occipital gyrus (CN: 0.55 ± 0.05, FES: 0.47 ± 0.05, *T*_78_ = 6.29, *P* < 0.001), left parahippocampal gyrus/amygdala/orbital frontal cortex (CN: 0.68 ± 0.05, FES: 0.62 ± 0.05, *T*_78_ = 4.66, *P* < 0.001). (See Fig. 2 and Table 2).

**Table 2:** Summary of the results obtained by One-way ANOVA analysis across the four study groups.

Anatomical area	Cluster-level		Peak-level		Effect size (Cohen’s d)					
	*P*_FWE-corr_	Size (voxels)	*F*	MNI coordinates of peak point	CN vs. FES	CN vs. UHR	CN vs. UFDR	FES vs. UHR	FES vs. UFDR	UHR vs. UFDR
L-Insula	0.047	756	10.2	(−43.5, 4.5, −3)	1.10***	1.04***	0.15	0.01	0.88*	0.83*
L-Inf Temporal/Fusiform	0.008	691	7.9	(−33, −10.5, −48)	0.98***	1.19***	0.80*	0.26	0.17	0.42
R-Inf Temporal/Fusiform	0.022	806	14.0	(34.5, −9, −49.5)	1.58***	0.01	0.71	1.19**	0.65	0.58
R-Mid/Sup Occipital	0.031	978	11.6	(24, −91.5, −18)	1.51***	1.08***	0.58	0.41	0.98*	0.54
L-Parahippocampal/Amygdala /Inf Frontal Orb	0.026	780	10.6	(−36, 9, −34.5)	1.13***	0.93**	0.35	0.17	1.44***	1.24***

Effect sizes with values greater than 0.8 are labeled in bold. Asterisk, ***P ≤ 0.001, **P ≤ 0.01, *P ≤ 0.05. Inf Temporal, inferior temporal gyrus. Mid/Inf Occipital, Middle/ Inferior occipital gyrus. Inf Frontal Orb, orbital frontal cortex.

We observed that multiple brain regions exhibited a monotonically decreasing trend of GMV from CN to UFDR to UHR to FES. Compared with the CN subjects, the UHR subjects exhibited similar GMV decline to FES subjects in the left insula (UHR: 0.60 ± 0.06, *T*_75_ = 4.15, *P* < 0.001), the visual processing areas including the right middle/superior occipital gyrus (UHR: 0.49 ± 0.05, *T*_75_ = 4.43, *P* < 0.001), the left inferior temporal/fusiform gyrus (UHR: 0.66 ± 0.07, *T*_75_ = 4.91, *P* < 0.001), the limbic system including the left parahippocampal gyrus/amygdala/orbital frontal cortex (UHR: 0.63 ± 0.06, *T*_75_ = 3.77, *P* = 0.002). Cohen’s d analysis also indicated marked GMV differences between the UHR and CN groups in these regions of interest (Cohen’s d: 0.93–1.19). On the other hand, the UHR subjects only had significantly higher GMV than the FES patients in the right inferior temporal gyrus/fusiform gyrus (UHR: 0.75 ± 0.12, *T*_47_ = 4.48, *P* < 0.001, Cohen’s d: 1.19). (See Fig. 2 and Table 2).

In contrast, the UFDR subjects only showed significant GMV decline compared to the CN subjects in the left inferior temporal/fusiform gyrus (UFDR: 0.69 ± 0.07, *T*_70_ = 2.96, *P* = 0.022), but exhibited attenuated abnormalities (Cohen’s d: 0.80). The UFDR subjects had significantly higher GMV than the FES patients in the left insula (UFDR: 0.65 ± 0.07, *T*_42_ = 2.88, *P* = 0.029, Cohen’s d: 0.88), the right middle/superior occipital gyrus (UFDR: 0.52 ± 0.04, *T*_42_ = 3.02, *P* = 0.019, Cohen’s d: 0.98) and the left parahippocampal gyrus/amygdala/orbital frontal cortex (UFDR: 0.70 ± 0.06, *T*_42_ = 4.77, *P* < 0.001, Cohen’s d: 1.44). Direct comparisons between the UHR and UFDR subjects revealed group differences in the left insula (*T*_39_ = 2.77, *P* = 0.039, Cohen’s d: 0.83) and left parahippocampal gyrus/amygdala/orbital frontal cortex (*T*_39_ = 4.10, *P* < 0.001, Cohen’s d: 1.24), where GMV was significantly reduced in the UHR group. (See Fig. 2 and Table 2).

**Fig. 3:**
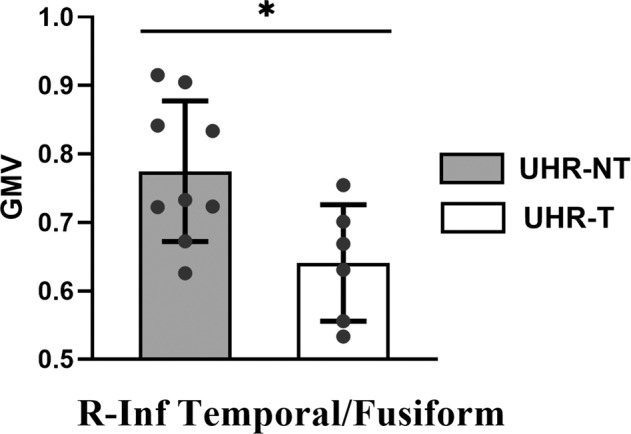
GMV differences between the UHR-T and UHR-NT subgroups. UHR-T and UHR-NT: transformed and non-transformed subgroups within the UHR group. Asterisk, *P ≤ 0.05. Inf Temporal, inferior temporal gyrus.

We further carried out a pilot experiment to test whether GMV abnormalities were more severe in UHR individuals who later developed psychosis. Among the UHR individuals, 15 subjects had 4-year longitudinal follow-up data, of which 6 UHR subjects were transformed into psychosis (UHR-T) and 9 were not transformed (UHR-NT). We found that the UHR-T subjects had significant lower GMV than the UHR-NT subjects in the right inferior temporal/fusiform gyrus (UHR-T: 0.64 ± 0.09, UHR-NT: 0.77 ± 0.10, *T*_13_ = 3.09, *P* = 0.03). (See Fig. 3).

### Correlations between symptom severity and GMV

**Fig. 4:**
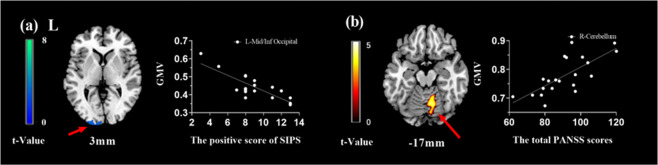
Correlations between symptom severity and GMV. The correlations were thresholder at *P*_voxel-wise_ < 0.005 (uncorrected), then corrected for multiple comparisons to *P*_FWE_ < 0.05 by using a cluster threshold. **a** Correlations between GMV and the positive score of SIPS in UHR group (*T*_peak-level_ = 6.28, *P*_FWE-corr_ = 0.013, *k* > 1052 or 3550.5 mm^3^). **b** Correlations between GMV and the total PANSS scores in FES group (*T*_peak-level_ = 4.91, *P*_FWE-corr_ = 0.041, *k* > 855 or 2,885.625 mm^3^). Mid/Inf Occipital, Middle/Inferior occipital gyrus. Mid/Inf Temporal, Middle/Inferior Temporal gyrus.

We performed exploratory correlation analyses in whole brain voxel-wise level to give a full description of possible symptom-related GMV alteration (*P*_voxel-wise_ < 0.005, uncorrected; FWE corrected in cluster level). We found that concurrent positive symptom on SIPS was negatively correlated with the GMV of the left middle/inferior occipital gyrus across the UHR subjects (*T*_peak-level_ = 6.28, *P*_FWE-corr_ = 0.013, *k* > 1052 or 3550.5 mm^3^; see Fig. 4a). For the FES subjects, the total score of PANSS was positively correlated with the GMV of the cerebellum (*T*_peak-level_ = 4.91, *P*_FWE-corr_ = 0.041, *k* > 855 or 2,885.625 mm^3^; see Fig. 4b). However, in this study, GMV did not show significant correlation with PANSS subscores (positive and negative) in the FES group or negative score of SIPS in the UHR group.

### The effect of confounding variables

We tested whether the GMV abnormalities were related to age, duration of illness, medication status, and/or dose. We first performed correlation analyses for each group separately to assess the relationships between age and GMV in the identified brain regions from the ANOVA model (see Supplementary Material Fig. S1). This analysis revealed no significant association, although there was a modest trend in the right middle/superior occipital gyrus in the UHR group (*R* = 0.47, *P* = 0.02, uncorrected). Then, we tested whether GMV differed as a function of medication or illness duration in the FES group (see Supplementary Material Table S1). We did not observe significant GMV differences between the medicated and unmedicated patients in the FES group (absolute *T* values < 1.77, *P* > 0.09). Moreover, GMV in the identified brain regions did not correlate with medication dose (absolute *R* values < 0.23, *P* > 0.50) or illness duration (absolute *R* values < 0.29, *P* > 0.18) among FES patients. These secondary analyses ruled out the possibility that the observed GMV abnormalities were driven by confounding variables such as age, medication, or illness duration.

## Discussion

The current study sought to disentangle the convergent and divergent pattern of gray matter alterations between the UFDR and UHR subjects. To our knowledge, this study is among the very few reports which directly compared GMV deficits and regional dysfunction between familial and clinical risk subjects for schizophrenia. We found that multiple brain regions exhibited a monotonically decreasing trend of GMV from CN to UFDR to UHR to FES. Compared with the CN subjects, the UHR subjects exhibited similar GMV decline to FES subjects in the bilateral insula, the visual processing areas and the limbic system. On the other hand, the UFDR group was only associated with focal GMV abnormalities in the inferior temporal gyrus and fusiform gyrus. Our results suggest that GMV deficits in schizophrenia are evident prior to the onset of full-blown illness and are more prominent in the UHR than UFDR individuals.

In this study, we found that the UHR subjects had GMV deficits in distributed brain regions similar to the changes in FES, mainly including the bilateral insula, the cortex of the visual pathway, and the limbic system. This finding was consistent with previous structural image studies of FES or UHR^8,14,30,31^. Among them, the insula plays an integrative role in salience processing across multiple sensory and cognitive domains^32^, and also links external and self-generated information from diverse functional systems^33^. Moreover, the insula is a key target for many psychiatric disorders in addition to schizophrenia^32,34,35^. Previous studies have also found that the GMV of the bilateral insula in the UHR subjects has a significant downward trend compared with CN, and shows a progressive change during the transition to psychosis^30^. It is proposed that the abnormalities of insular function in schizophrenia patients mainly include the processing of visual and auditory emotional information and neuronal representations of the self^36^. In our study, the UHR subjects’ main diagnostic criteria were a positive symptom profile including delusions and hallucinations. Delusions are the result of cognitive efforts to understand aberrantly salient experiences, while hallucinations are direct experiences with the aberrant salience of internal representations^32^. This indicates that insular dysfunction may impair the UHR individuals’ ability to distinguish between self-generated information and external information, leading to the appearance of core positive symptoms of schizophrenia^32,33^.

On the other hand, in the whole UHR group, the right inferior temporal/fusiform gyrus did not show a similar GMV decline as the left homotopic region. However, our pilot experiment revealed that the UHR-T subjects had significant lower GMV than the UHR-NT subjects in the right inferior temporal/fusiform gyrus (see Fig. 3). This may indicate that the structural changes of the temporal region have left-right asymmetry in the UHR subjects, which is consistent with previous studies^37,38^. Previous cross-sectional and longitudinal studies have collectively suggested that GMV decline is more severe in the UHR-T group compared with the UHR-NT group^14,39^. Therefore, baseline GMV abnormalities in the right temporal regions may increase the risk of psychopathology in the UHR population, and serve as a landmark brain region for developing psychosis. This provides structural evidence for the possibility that information perception dysfunction may serve as risk factors for susceptibility to psychosis.

Another major finding of this study is that the UHR subjects showed GMV decreases in the cortex of the ventral visual pathway, which mainly includes the primary visual cortex of the occipital lobe and the inferior temporal gyrus projected downstream^40^. The emphasizing of lower-level sensory systems is contrary to the frequent highlight on deficits of higher-order cognitive function in schizophrenia. Studies have confirmed that the impairments of basic visual processing, including motion and color perception, perceptual organization and scan paths and so on, are important parts of the characteristics of schizophrenia^41^. Several studies have also reported the structural alterations of the occipital and inferior temporal gyrus in high-risk populations of schizophrenia^14,42,43^.

In this study, we further found that the GMV of the occipital lobe in the UHR subjects was negatively correlated with the severity of the positive symptoms (see Fig. 4a). It suggests a structural basis for the changes in symptoms of UHR population before the onset of the disease, and as the course of the disease prolongs and the symptoms worsen, the visual processing area is more prone to abnormalities. Our study also found decreased GMV in several limbic brain regions in the UHR subjects, of which the parahippocampus and amygdala are downstream projections of the ventral visual pathway^44,45^, and the orbitofrontal cortex is involved in integrating emotional information from the sensory and limbic systems^46^. Therefore, our results may also implicit that UHR population have developed a structural basis for impaired visual-related emotional processing abnormalities^47,48^.

Interestingly, we also found that the UFDR subjects showed GMV abnormalities in the temporal lobe part of the ventral visual pathway. However, the UFDR subjects had fewer abnormal brain regions than the UHR subjects, only appeared in the inferior temporal gyrus and fusiform gyrus. Our findings are consistent with a longitudinal study by Job et al., who found that among all brain regions, GMV in the inferior temporal gyrus had the highest accuracy in predicting the onset and non-onset of schizophrenia in individuals at high genetic risk for schizophrenia, suggesting that the inferior temporal gyrus was the brain region most affected by genetic susceptibility^49,50^. Kuroki et al. reported that smaller inferior temporal gyrus gray matter volumes may be related to pathology common to both schizophrenia and affective psychosis^51^. On the other hand, Goghari et al. have reported that reduced fusiform gray matter volumes in both schizophrenia patients and unaffected relatives, which maybe associated with facial emotion recognition deficits^52^. Therefore, our results suggest that visual perception deficiency may be both genetic and symptom risk factors for schizophrenia, and its abnormality degree in UHR population is more serious than that in unaffected genetic risk population. It should also be noted that our genetic high-risk group inclusion criteria included only asymptomatic first-degree relatives. Therefore, the abnormal brain regions in the UFDR subjects in this study are more focal than those reported in other studies of genetically at-risk populations^22,53^.

In our study, the volume of the cerebellar part of the FES subjects was positively correlated with the total score of PANSS (see Fig. 4b), indicating that the greater GMV in the cerebellum, the more severe the patient’s psychotic symptoms. The role of the cerebellum in schizophrenia has been highlighted by Andreasen’s hypothesis of “cognitive dysmetria”, which suggests that dysfunctions in the cortico-cerebellar-thalamo-cortical circuit could explain a variety of behavioral symptoms of schizophrenia^54–57^. The correlation between the local GMV of cerebellum and schizophrenia symptoms provides further structural evidence for demonstrating that the abnormality of cerebellum is closely related to the physiopathology of schizophrenia. However, the current literature on structural brain abnormalities in schizophrenia with regard to cerebellum is inconsistent. For example, some previous literature reported a decline in cerebellar GMV^58,59^, while other independent studies reported cerebellar GMV increased in FES subjects^59,60^. It is hypothesized that neuron pruning defects may cause larger regional gray matter and more severe symptoms in schizophrenia patients^61^. However, the neural mechanisms underlying this phenomenon need to be explored further.

This study includes the following limitations. First, the sample size is relatively small, especially for UFDR and UHR subjects. Large samples and multi-center data are needed to further validate our findings. Second, there are significant differences in the ratio of males to females between the UFDR subjects and other groups. Several studies have reported gender differences in neuroanatomical and psychopathological symptoms in high-risk individuals and patients with schizophrenia^62–64^. We added gender as a covariate in the ANOVA analysis, which mitigated the possible influence of gender on the results. Third, we only used VBM to measure the morphological changes between the UHR and UFDR groups. Alterations of integrated networks of brain regions in different high-risk populations of schizophrenia need to be further explored by using multimodal MRI technologies.

In summary, our study showed a monotonic downward trend of GMV from CN to UFDR to UHR to FES in distributed brain regions, mainly in the insular lobe and the cortex of the visual pathway. In particular, both the UHR and UFDR subjects exhibited structural brain abnormalities in the visual processing areas. However, the UFDR subjects only involved part of the advanced processing areas of the visual pathway, while the UHR subjects were also related to the primary visual perception areas and other visual-related emotional processing areas. These results indicate that structural abnormalities have appeared before the onset of the two types of high-risk groups, and the UHR subjects with psychiatric symptoms are more serious and broader in brain area than the UFDR subjects with asymptomatic but genetic risk.

## Supplementary information

supplementary material

## Data Availability

The data that support the findings of this study are available from the corresponding author upon reasonable request.

## Code availability

We used standardized toolbox to analyze the data, no special code was used in data analysis.
