# Supplementary material for: Convergent and divergent gray matter volume abnormalities in unaffected first-degree relatives and ultra-high risk individuals of schizophrenia
Source: Schizophrenia (Heidelb). 2022 Jun 4;8(1):55. doi: 10.1038/s41537-022-00261-9 (PMC9261104; doi:10.1038/s41537-022-00261-9)
Supplement: Supplementary file 1 — supplementary material [file 41537_2022_261_MOESM1_ESM.docx]

**Supplementary Material**


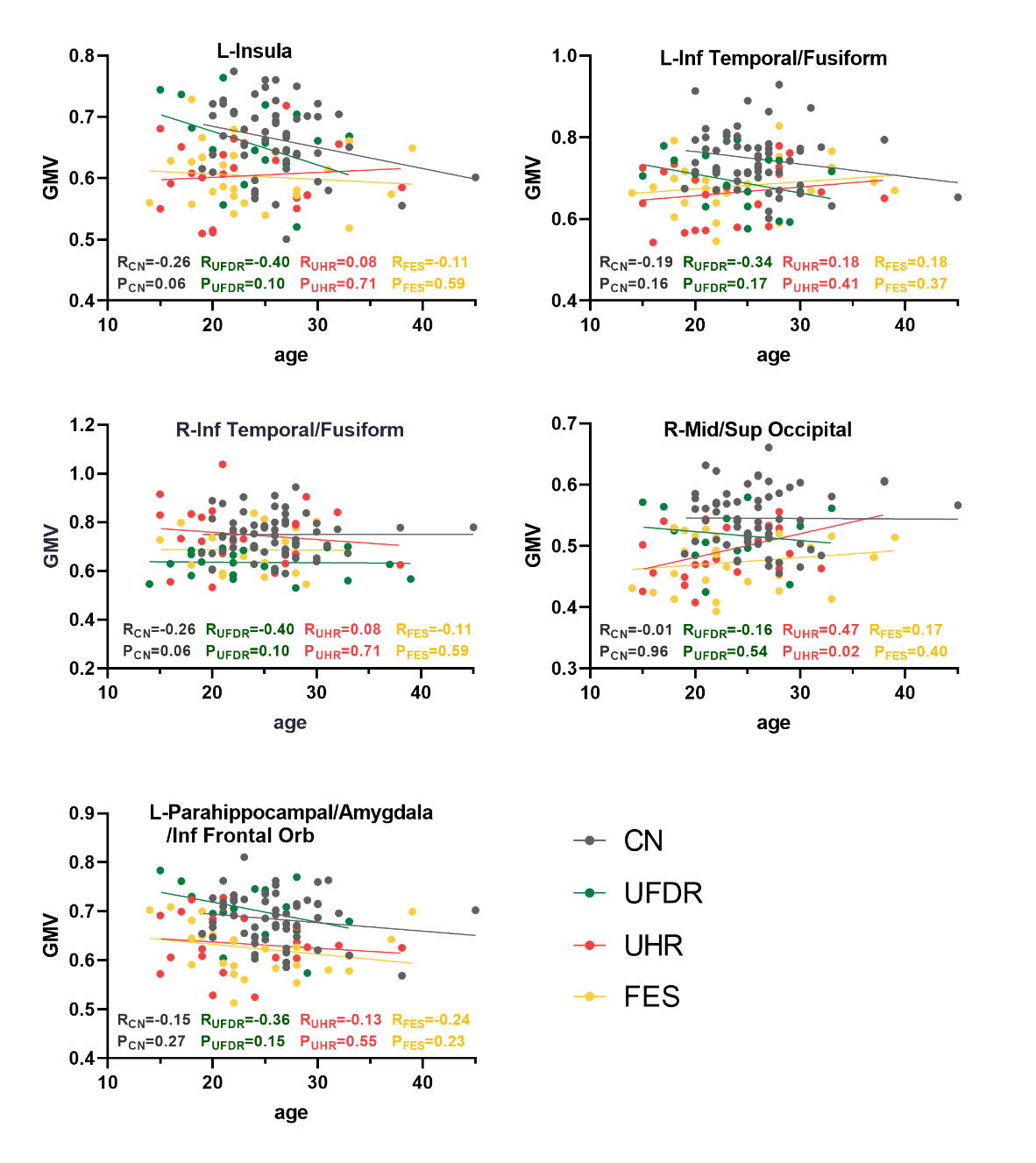


**Fig. S1 Correlations between age and GMV in the ROIs from ANOVA model.** Inf Temporal, inferior temporal gyrus. Mid/Inf Occipital, Middle/Inferior occipital gyrus. Inf Frontal Orb, orbital frontal cortex.

**Table S2. Analysis of the effect of medication and illness duration on GMV of ROIs in FES group**

|  | Correlation in FES | |  | T-test within FES |
| --- | --- | --- | --- | --- |
|  | Olanzapine equivalent dose (FES-M, n = 11) P (R) | Illness duration  (FES, n = 23) P (R) |  | FES-M (n = 11) vs.  FES-UM (n = 9)  P (T, Df) |
| L-Insula | 0.85 (0.06) | 0.18 (0.29) |  | 0.22 (-1.28, 18) |
| L-Inf Temporal/Fusiform | 0.61 (0.17) | 0.87 (-0.04) |  | 0.57 (-0.58, 18) |
| R-Inf Temporal/Fusiform | 0.50 (0.23) | 0.75 (0.07) |  | 0.95 (0.06, 18) |
| R-Mid/Sup Occipital | 0.61 (-0.17) | 0.48 (-0.15) |  | 0.22 (-1.26, 18) |
| L-Parahippocampal/Amygdala /Inf Frontal Orb | 0.65 (-0.15) | 0.71 (-0.08) |  | 0.99 (0.01, 18) |

Medication and illness duration information were not available for 6 and 3 FES subjects, respectively. FES-M and FES-UM: medicated and unmedicated subgroups within the FES group.
